# Supplementary material for: How Sure is the Driver? Modelling Drivers’ Confidence in Left-Turn Gap Acceptance Decisions
Source: Comput Brain Behav. 2024 Jul 12;7(3):437–56. doi: 10.1007/s42113-024-00207-7 (PMC13298634; doi:10.1007/s42113-024-00207-7)
Supplement: Supplementary file 1 — Supplementary file1 (PDF 844 KB) [file 42113_2024_207_MOESM1_ESM.pdf]

Supplementary information for "Are you sure? Modelling the Confidence of a Driver in Left-Turn Gap Acceptance Decisions"

Floor Bontje[1], Arkady Zgonnikov [1,2]

This supplementary information document provides the following appendices: A) figures of the mixed-effects models of decision behavior, response time and confidence; B) an overview of the random effects terms present in the mixed-effects models; C) an overview of the correlations between the response time and the initial throttle operation moment, between confidence and response time and between confidence and the initial throttle operation moment; D) an explanation of the found relations related to the initial throttle operation moment; E) the results of the mixed-effects analyses describing the relation between confidence on the one hand and the velocity and distance to the centre of the intersection on the other (the action dynamics); F) an description of the optimisation of model parameters; and G) an statement about the excluded left-turns.

Appendix A - Figures

Decision behavior

$$decision \sim distance + TTA + (1|ID)$$

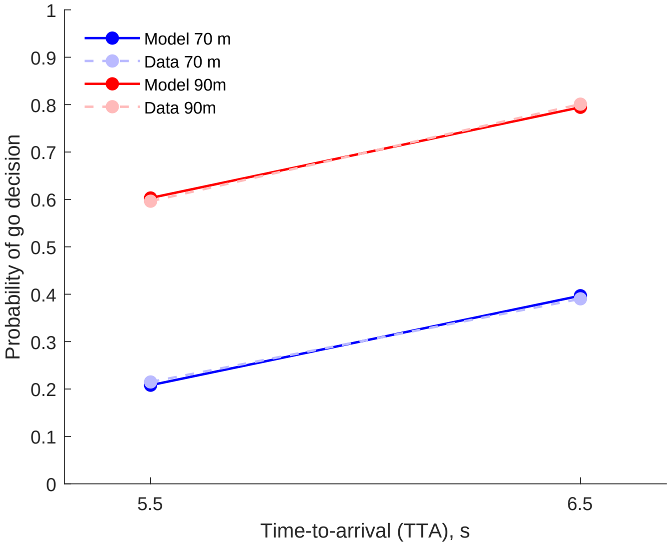

Fig. 1: Mixed-effects model of the probability of making "go" decisions.

[1] *Department of Cognitive Robotics, Faculty of Mechanical, Maritime and MaterialsEngineering, Delft University of Technology, Netherlands*
[2] *AiTech, Delft University of Technology, Netherlands*

Decision response time

$$RT \sim distance * decision + TTA * decision + (decision|ID)$$

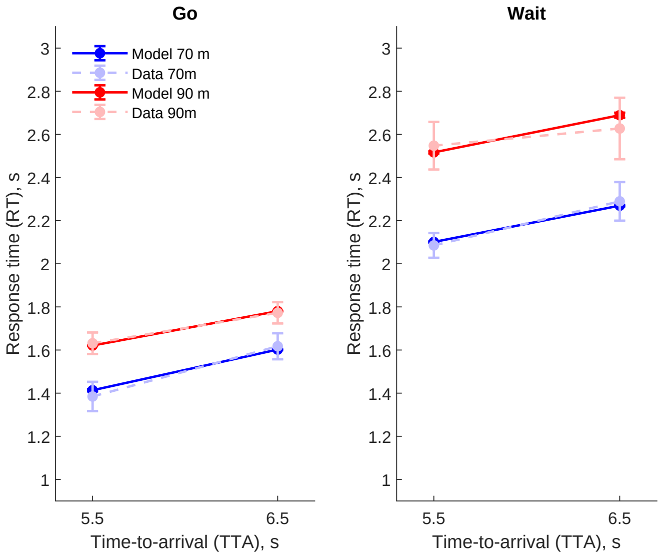

Fig. 2: Linear mixed-effects model of the response time.

Confidence

$$Confidence \sim RT * decision + distance * decision + TTA * decision + (decision|ID)$$

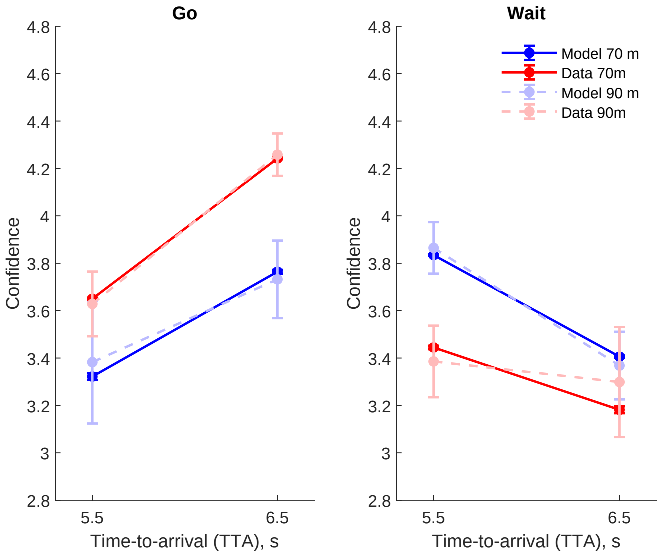

Fig. 3: Linear mixed-effects model of confidence based on the response time, distance, time-to-arrival and decision.

$$Confidence \sim Thr_{int} * decision + distance * decision + TTA * decision + (decision|ID)$$

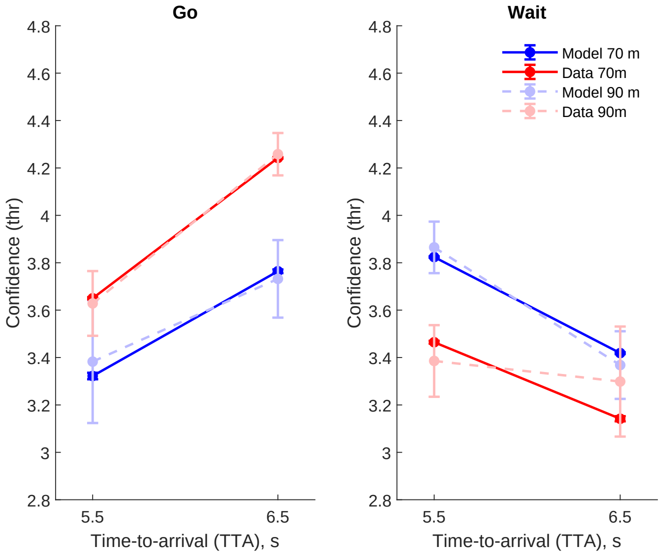

Fig. 4: Linear mixed-effects model of confidence based on the initial throttle operation moment, distance, time-to-arrival and decision.

Appendix B - Random Effects

In order to account for the between-participants differences, in the analysis of the relationships between the dependent and independent variables using mixed-effects models, we used random intercepts or slopes per participant (ID). For these analysis we use a significance level of 0.05.

Decision behavior

We investigated the influence of the distance and time-to-arrival (TTA) conditions on the decision outcome (the probability of a "go" decision) by using the following mixed-effect model: $decision \sim distance + TTA + (1|ID)$
The random intercept $(1|ID)$ describing the influence of individual differences has a standard deviation (SD) 0.3978 of and 95% confidence interval (CI) of $[0.3839, 0.4122]$. We found for ten out of the seventeen participants an additional significant intercept (Table 1).

Estimate	pValue
0.13796	0.034472
-0.24048	0.00023872
0.13491	0.037971
-0.21088	0.0013205
0.42177	1.2186e-10
-0.26047	6.7498e-05
-0.15967	0.01408
0.1417	0.033149
-0.42763	1.1548e-09
0.34158	1.7393e-07

TABLE 1: Estimated values and pValues of the significant random intercepts ($n = 17$) in the decision behavior mixed-effects model.

Response time

We investigated the effect of the TTA and distance conditions with the following linear mixed-effects model, $RT \sim distance * decision + TTA * decision + (decision|ID)$.
The model accounts for individual differences with the random effects, consisting of a random intercept and a random decision slope (i.e. an additional term for "wait" decisions) (Table 2).

	SD	95% CI
Intercept	0.25022	[0.16431, 0.38105]
Wait decision	0.34325	[0.22103, 0.53306]

TABLE 2: The standard deviation (SD) and the 95% confidence interval (CI) of the found random effects in the linear mixed-effects model of the response time (RT).

We found a significant random effect of the intercept for six participants (Table 3) and an additional random effect for the "wait" decision for seven participants (Table 4).

Estimate	pValue
-0.32815	0.0051979
0.37553	0.0020354
-0.27047	0.021912
0.74597	1.1226e-09
-0.29145	0.012199
0.7309	0.0013217

TABLE 3: Estimated values and pValues of the found ($n = 6$) significant random effect intercepts in the response time linear mixed-effects model.

Estimate	pValue
0.51067	1.2307e-09
-0.5062	1.2217e-07
0.20642	0.015385
-0.23606	0.0018559
-0.25012	0.0022344
0.18155	0.032157
-0.25454	0.00091624

TABLE 4: Estimated values and pValues of the found ($n = 7$) significant random effect slopes for "wait" decisions in the response time linear mixed-effects model.

Confidence (RT)

We investigated the effects of the response time, TTA and distance on confidence with the following linear mixed-effects model: $Confidence \sim RT * decision + TTA * decision + distance * decision + (decision|ID)$
The model accounts for individual differences with random effects, consisting of a random intercept and a random slope (Table 5).

	SD	95% CI
Intercept	0.51158	[0.34788, 0.75232]
Wait decision	0.48767	[0.31956, 0.74422]

TABLE 5: The standard deviation (SD) and the 95% confidence interval (CI) of the found random effects in the linear mixed-effects model of confidence (response time).

We found a significant random intercept for five participants (Table 6) and an additional random slope in "wait" decisions for nine participants (Table 7).

Estimate	pValue
-0.42034	0.038575
-0.56695	0.004159
0.63415	0.0014371
1.0984	6.1472e-08
-0.61043	0.009719

TABLE 6: Estimated values and pValues of the found ($n = 5$) significant random intercept in the confidence (response time) linear mixed-effects model.

Confidence (throttle)

We investigated the effects of the first moment in time of using the throttle (Thr_{int}), TTA and distance

Estimate	pValue
0.66059	0.00014618
-0.49468	0.0026842
-0.58171	0.00071363
0.35686	0.039236
0.97672	2.7953e-10
-0.67047	5.7792e-05
-0.51608	0.010064
-0.6033	0.00097385
0.54496	0.0014252

TABLE 7: Estimated values and pValues of the found ($n = 9$) significant random addition slope for "wait" decisions in the confidence (response time) linear mixed-effects model.

on confidence with the following linear mixed-effects model: $Confidence \sim Thr_{int} * decision + TTA * decision + distance * decision + (decision|ID)$
The model accounts for individual differences with random effects, consisting of a random intercept and a random slope (Table 8).

	SD	95% CI
Intercept	0.45881	[0.31474, 0.66882]
Wait decision	0.49196	[0.32783, 0.73825]

TABLE 8: The standard deviation (SD) and 95% confidence interval (CI) of the random effects for the linear mixed-effects model of confidence (throttle).

We found a significant random intercept for four participants (Table 9) and a random slope in "wait" decisions for nine participants (Table 10).

Estimate	pValue
-0.6084	0.003291
0.75733	0.00024556
1.1477	3.4713e-08
-0.62541	0.010011

TABLE 9: Estimated values and pValues of the found ($n = 4$) significant random intercept in the confidence (throttle) linear mixed-effects model.

Estimate	pValue
0.51859	0.0054811
-0.57188	0.00063699
0.34505	0.039499
0.94403	4.177e-10
-0.54311	0.00075716
-0.58911	0.0027873
-0.4385	0.013682
-0.3781	0.016327
0.53283	0.001304

TABLE 10: Estimated values and pValues of the found ($n = 9$) significant random addition slope for "wait" decisions in the confidence (throttle) linear mixed-effects model.

APPENDIX C - CORRELATIONS

In the research, several potential correlations were investigated:

1) Decision response time – Initial throttle operation moment
2) Confidence – Decision response time
3) Confidence – Initial throttle operation moment

Decision response time - Initial throttle operation moment

Decision	r	pValue
All	0.2332	2.0647e-20
Go	0.2818	1.6121e-15
Wait	-0.2409	1.4624e-11

TABLE 11: Correlation coefficients (r) between decision response time (RT) and the initial throttle operation moment.

Confidence - Response time

Decision	r	pValue
All	-0.2729	1.2874e-27
Go	-0.2114	3.1298e-09
Wait	-0.2490	2.8442e-12

TABLE 12: Correlation coefficients (r) between confidence and the response time (RT).

TTA\distance	70 m	90 m
5.5 seconds	-0.49 ($p = 4.13e-06$)	-0.34 ($p = 2.5e-07$)
6.5 seconds	-0.22 ($p = 0.0062$)	-0.35 ($p = 2.5e-10$)

TABLE 13: Correlation coefficients (r) between response time and confidence judgments – for different traffic conditions described by the time-to-arrival (TTA) and distance gap conditions in ”go” decisions.

TTA\distance	70 m	90 m
5.5 seconds	-0.23 ($p = 6.4e-05$)	-0.24 ($p = 0.0028$)
6.5 seconds	-0.19 ($p = 0.0038$)	-0.13 ($p = 0.27$)

TABLE 14: Correlation coefficients (r) between response time and confidence judgments – for different traffic conditions described by the time-to-arrival (TTA) and distance gap conditions in ”wait” decisions.

Confidence - Initial throttle operation moment

Decision	r	pValue
All	-0.2224	1.1645e-18
Go	-0.2735	1.1195e-14
Wait	-0.1591	9.8448e-06

TABLE 15: Correlation coefficients (r) between confidence and the initial throttle operation moment.

TTA\distance	70 m	90 m
5.5 seconds	-0.44 ($p = 3.3e-05$)	-0.46 ($p = 4.0e-13$)
6.5 seconds	-0.37 ($p = 2.5e-06$)	-0.27 ($p = 1.5e-06$)

TABLE 16: Correlation coefficients (r) between initial throttle operation moment and confidence judgments – for different traffic conditions described by the time-to-arrival (TTA) and distance gap conditions in ”go” decisions.

TTA\distance	70 m	90 m
5.5 seconds	-0.055 ($p = 0.35$)	-0.089 ($p = 0.28$)
6.5 seconds	-0.21 ($p = 0.0014$)	-0.32 ($p = 0.0040$)

TABLE 17: Correlation coefficients (r) between initial throttle operation moment and confidence judgments – for different traffic conditions described by the time-to-arrival (TTA) and distance gap conditions in ”wait” decisions.

Appendix D - Initial throttle Operation Moment

In this research we used a button press to measure the response time. A more natural way of measuring response times, used in previous research with a similar task, is by using the timing of gas throttle depression as indication of the decision moment [1]. To get a better understanding of the initial use of the throttle we investigated the relation between the initial throttle operation moment at the one hand hand and the response time and confidence at the other. We firstly assessed the relation between the indicated response time (button press) and the initial throttle operation moment. Secondly, we assessed the relation between confidence and the initial throttle operation moment. We hypothesise that the initial throttle operation moment is related to the response time and that it can be used as a behavior measure of the response time for "go" decisions. Moreover, we expect a negative relation between the initial throttle operation moment and confidence. This expectation is a result of the combination of our expectation that the initial throttle operation moment is a measure of the decision response time and the found negative relation between the indicated decision response time and confidence. With the use of correlations we investigated 1) the relation between the initial throttle operation moment and response time and, 2) the relation between the initial throttle operation moment and confidence. Moreover, we used linear mixed-effects models to describe the relation between confidence on the one hand and the time-to-arrival, the distance gap and the initial throttle operation moment on the other. $Confidence \sim Thr_{int} * decision + distance * decision + TTA * decision + (decision|ID)$.

We investigated the relation between the response time and the initial throttle operation moment to find out if the initial throttle operation moment can be used as behavior-based indication of response time. We found, contradictory to our hypothesis, that in this task the initial throttle operation moment cannot be considered as a "clear" behavior-based indication of the response time. We observed a moderate positive correlation between the response time and initial throttle operation moment for "go" decisions (r = 0.28, p = 1.6e-15) and the moderate negative correlation for "wait" decisions (r = -0.24, p = 1.46e-11). Moreover, we observed that participants started driving before indicating their decision in 47% of all the "go" and in 27% of all the "wait" decisions (Figure 5).

Regarding the relation between the initial throttle operation moment and confidence, we observed a negative correlation (r = -0.22, p = 1.16e-18) between confidence and the initial throttle operation moment over all decisions. The results of the linear regression analysis (Table 18) demonstrated a negative relation between the initial throttle operation moment and confidence (b = -0.34, t = -5.1, p = 4.1e-07) for "go" decisions. For "wait" decisions, we found a relatively positive fixed effect (b = 0.33, t = 4.7, p = 3.3e-06) of the initial throttle operation moment on confidence compared to "go" decisions. This additional effect levelled out the effect of the initial throttle operation moment on confidence found for "go" decisions (b = -0.34, F = 213, p = 3.3e-45), resulting in a net effect of -0.015 (F = 226, p = 1.2e-47) in "wait" decisions. This indicates that the effect of the initial throttle operation moment on confidence is strongly reduced for "wait" decisions as one may expect. That is because for "go" decisions the execution of the decision starts with entering the intersection by operating the gas throttle, while for "wait" decisions the execution of the decision means remaining in the same position on the road.

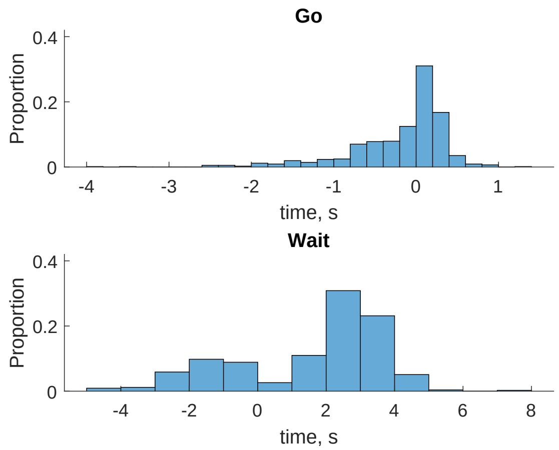

Fig. 5: Distribution of response time (button press) relative to the the initial throttle operation moment, for "go" and "wait" decisions separately.

	Estimate	Std. Error	t-score	pValue
Intercept	-2.406	0.5163	-4.660	3.445e-06
TTA	0.6925	0.06356	10.90	1.125e-26
Distance	0.02978	0.003414	8.722	6.993e-18
Thr_{int}	-0.3413	0.0671	-5.0871	4.086e-07
Wait decision	10.32	0.7073	14.58	3.325e-45
Wait decision: TTA	-1.147	0.09024	-12.71	2.839e-35
Wait decision: Distance	-0.05141	0.004853	-10.60	2.319e-25
wait decision: Thr_{int}	0.3260	0.06981	4.670	3.273e-06

TABLE 18: Results of regression analysis of the effect of the initial throttle operation moment (Thr_{int}), the distance gap and TTA conditions on confidence judgments for different decision outcomes. Linear mixed-effects model: $Confidence \sim Thr_{int} * decision + TTA * decision + distance * decision + (decision|ID)$.

APPENDIX E - ACTION DYNAMICS

We investigated the effect of confidence on the two used measures of action dynamics, velocity profile and distance to centre of intersection, with use of linear mixed-effects models defined in general by: $Metric \sim Confidence * decision + (1|ID)$.
For both measures we took four different metrics into account: maximum/minimum value, deviation from the individual mean, deviation from the group mean and the RMSD.

Velocity

Maximum velocity

	Estimate	SD Error	t-score	pValue
Intercept	9.9337	0.44954	22.097	3.8434e-94
Confidence	0.097675	0.080369	1.2153	0.22442
Decision wait	-0.26592	0.40842	-0.65111	0.51508
Decision wait: conf.	-0.13321	0.10769	-1.237	0.21629

TABLE 19: Fixed coefficients of the linear mixed-effects model describing the relation between confidence and the maximum value of the velocity profile.

	SD	95% CI
Intercept	1.3148	[0.92872, 1.8612]

TABLE 20: Random effects of the linear mixed-effects model describing the relation between confidence and the maximum value of the velocity profile.

Deviation from the individual mean

This model has a Hessian matrix with NaNs or Infs, which indicates that the model has more covariance parameters than supported by the data.

	Estimate	SD Error	t-score	pValue
Intercept	-0.18554	0.16697	-1.1112	0.26666
Confidence	0.047861	0.04165	1.1491	0.25069
Decision wait	0.051122	0.22532	0.22689	0.82054
Decision wait: conf.	-0.01007	0.058309	-0.1727	0.86291

TABLE 21: Fixed coefficients of the linear mixed-effects model describing the relation between confidence and the deviation from the individual mean of the distance to the velocity profile.

	SD	95% CI
Intercept	2.6211e-16	[NaN, NaN]

TABLE 22: Random effects of the linear mixed-effects model describing the relation between confidence and the deviation from the individual mean of the distance to the velocity profile.

Deviation from the group mean

	Estimate	SD Error	t-score	pValue
Intercept	-0.66223	0.29141	-2.2725	0.023197
Confidence	0.10768	0.048922	2.2011	0.027879
Decision wait	0.75535	0.24851	3.0396	0.0024095
Decision wait: conf.	-0.092263	0.065537	-1.4078	0.15939

TABLE 23: Fixed coefficients of the linear mixed-effects model describing the relation between confidence and the deviation from the group mean of the velocity profile.

	SD	95% CI
Intercept	0.90075	[0.63756, 1.2726]

TABLE 24: Random effects of the linear mixed-effects model describing the relation between confidence and the deviation from the group mean of the velocity profile.

RMSD

	Estimate	SD Error	t-score	pValue
Intercept	0.98234	0.16598	5.9184	4.0042e-09
Confidence	-0.060233	0.035916	-1.6771	0.093731
Decision wait	0.5175	0.1831	2.8263	0.0047707
Decision wait: conf.	0.0314	0.048229	0.65106	0.51511

TABLE 25: Fixed coefficients of the linear mixed-effects model describing the relation between confidence and the RMSD of the distance to the velocity profile.

	SD	95% CI
Intercept	0.35621	[0.24773, 0.5122]

TABLE 26: Random effects of the linear mixed-effects model describing the relation between confidence and the RMSD of the distance to the velocity profile.

Distance to the centre of the intersection

Minimum distance

	Estimate	SD Error	t-score	pValue
Intercept	2.6499	0.19741	13.423	6.388e-39
Confidence	-0.16805	0.04266	-3.9393	8.5385e-05
Decision wait	-0.72751	0.21748	-3.3452	0.00084219
Decision wait: conf.	0.19404	0.057285	3.3874	0.0007236

TABLE 27: Fixed coefficients of the linear mixed-effects model describing the relation between confidence and the minimum value of the distance to the centre of the intersection.

	SD	95% CI
Intercept	0.42517	[0.29627, 0.61017]

TABLE 28: Random effects of the linear mixed-effects model describing the relation between confidence and the minimum value of the distance to the centre of the intersection.

Deviation from the individual mean

This model has a Hessian matrix with NaNs or Infs, which indicates that the model has more covariance parameters than supported by the data.

	Estimate	SD Error	t-score	pValue
Intercept	0.16542	0.15874	1.0421	0.29755
Confidence	-0.042672	0.039598	-1.0776	0.28137
Decision wait	-0.26849	0.21422	-1.2534	0.21027
Decision wait: conf.	0.071649	0.055437	1.2925	0.1964

TABLE 29: Fixed coefficients of the linear mixed-effects model describing the relation between confidence and the deviation from the individual mean of the distance to the centre of the intersection.

	SD	95% CI
Intercept	0	[NaN, NaN]

TABLE 30: Random effects of the linear mixed-effects model describing the relation between confidence and the deviation from the individual mean of the distance to the centre of the intersection.

Deviation from the group mean

	Estimate	SD Error	t-score	pValue
Intercept	0.40604	0.22444	1.8091	0.070624
Confidence	-0.10937	0.044829	-2.4397	0.014813
Decision wait	-0.64016	0.2281	-2.8065	0.0050717
Decision wait: conf.	0.17602	0.06012	2.9279	0.0034634

TABLE 31: Fixed coefficients of the linear mixed-effects model describing the relation between confidence and the deviation from the group mean of the distance to the centre of the intersection.

	SD	95% CI
Intercept	0.57	[0.40107, 0.81009]

TABLE 32: Random effects of the linear mixed-effects model describing the relation between confidence and the deviation from the group mean of the distance to the centre of the intersection.

RMSD

	Estimate	SD Error	t-score	pValue
Intercept	1.4447	0.12608	11.459	3.1844e-29
Confidence	-0.074056	0.02703	-2.7397	0.00622
Decision wait	0.058083	0.13776	0.42162	0.67336
Decision wait: conf.	0.043074	0.094796	1.187	0.23543

TABLE 33: Fixed coefficients of the linear mixed-effects model describing the relation between confidence and the deviation from the group mean of the distance to the centre of the intersection.

	SD	95% CI
Intercept	0.27727	[0.19324, 0.39783]

TABLE 34: Random effects of the linear mixed-effects model describing the relation between confidence and the RMSD of the distance to the centre of the intersection.

Appendix F - Optimisation of Model Parameters

For optimisation of the model parameters, we made use of the "fmincon" function of MATLAB R2020a. The function searches for the set of parameters which result in a (local) minimum of the loss function of the performance of the model. It starts searching for an optimal set of parameters round an initial set of parameters. For this is the initial set of parameters defined in advance.

Loss functions

For the loss functions of the performance of the models, we made use of the weight least squares (WLS) and the root mean square error (RMSE) for respectively the decision and confidence models.

Decision models

To train the decision models, we made use of a newly defined loss function build up from the WLS of the model prediction. The WLS was calculated with the use of vincentized distributions [2]. For each of the four different conditions present in the experiment, the WLS was calculated, using two separate terms describing the WLS for "go" and "wait" decisions. The total sum of the WLS over all conditions was used as loss function.

Confidence models

For the confidence models, we used the root mean square error (RMSE) as loss function, which can be described by the following equations:

$$RMSE = \sqrt{\frac{\sum(Conf_{pr} - Conf_{expr})^2}{N_conf}}$$

$$Conf_i = [\mu_{conf,go,i}, \mu_{conf_wait,i}]$$

$\mu_{conf,decision,i}$ contains the mean confidence values for the four conditions in the specified decision of the prediction or the experiment. N_{conf} is the number of measuring points, so $N_{conf} = 4 * 2 = 8$.

Parameters

Decision model

The initial set of parameters used to train the decision models was obtained with the use of the optimisation code "03_fit_model.py" which accompanied our baseline decision model [1]. This optimisation was focused on the prediction of left-turn gap acceptance decision behavior by a dynamic drift-diffusion model (DDM) only accounting for "go"-decisions. This set of initial parameters was as a result not able to predict the response times in "wait" decisions accurately (figure 6). In order to describe both the response times in "go" and "wait" decisions we used the newly defined loss function. This new loss function was used in combination with the initially found parameters to find the parameter set describing both "go" and "wait" decisions for a dynamic DDM and for a race model, Table 35.

	WLS	α	β	b_0	k	μ_{ND}	σ_{ND}	θ_{crit}
Initial	3.08	0.985	0.101	1.14	0.357	1.40	0.117	13.7
Trained	1.53	1.12	0.109	1.41	0.396	1.51	0.140	14.0

TABLE 35: *Initial* DDM model (focus on "go" decisions) and *trained* DDM model (focus on "go" and "wait" decision): a) comparison of performance of the decision models using the mean WLS over 20 models, b) the parameter values found through optimisation.

The results of the parameter optimisation for a race model, describing the cognitive process by two competing decision variables, Table 36 showed that the race model with decision dependent parameters had an improved performance (lowest WLS-values). A high amount of decision dependent parameters (> 2) resulted in the best performances however this comes with higher risk of over fitting. Therefor, that is why we chose to only consider the models with a maximum of 2 decision dependent parameters.

These results are based on the initial parameter set (Table 35) of the DDM which was only able to predict "go" decisions not able to predict both "go" and "wait" decisions. Therefore, we additionally investigated whether the use of the optimal parameters for the DDM describing "go" and "wait" decisions could improve the optimisation of the race models. We found that the performance of the race model with the decision dependent drift rate parameter and decision dependent critical value improved (Table 37).

Confidence models

The confidence models presented in this research are built on decision models by adding decision dependent sensitivity and bias parameters and by potentially allowing for additional evidence accumulation.

Bias and sensitivity parameters

We found the values of the bias (c_0) and sensitivity (c) parameters (Table 38) using a linear regression model of the relation between confidence and the input value (V_c) in combination with the "fmincon" function. We used the "fmincon" function to constraint the sensitivity parameters to be equal or larger than zero.

Inter-judgment times

We allowed for post decision evidence accumulation in two confidence models. The additional evidence accumulation time, inter-judgment time (τ), could not be measured during the experiment thus had to be modelled. We calculated the effect of the different values of the inter-judgment time on the RMSE to do so. Over a time scale from

	WLS	α	β	b_0	k	μ_{ND}	σ_{ND}	θ_{crit}
race (α)	2.30	Go: 1.18 Wait: 1.18	0.107	1.26	0.448	1.59	0.131	13.7
race (α & b_0)	2.37	Go: 0.819 Wait: 0.826	0.0934	Go: 0.807 Wait: 0.500	0.306	1.68	0.101	12.4
race (α, b_0, k)	1.45	Go: 1.20 Wait: 1.14	0.100	Go: 1.22 Wait:1.35	Go: 0.398 Wait: 0.438	1.49	0.120	13.3
race (α, b_0, k, θ_{crit})	1.77	Go: 1.10 Wait: 1.09	0.110	Go: 1.23 Wait: 1.24	Go: 0.378 Wait: 0.377	1.39	0.124	Go: 13.82 Wait: 13.80
race (α & θ_{crit})	2.04	Go: 1.53 Wait: 1.49	0.110	1.28	0.331	1.50	0.112	Go: 13.8 Wait: 13.9
race (all)	0.86	Go: 1.11 Wait: 1.04	Go: 0.103 Wait: 0.101	Go: 1.20 Wait: 1.21	Go: 0.379 Wait: 0.341	Go: 1.401 Wait: 1.58	Go: 0.128 Wait: 0.131	Go: 13.4 Wait: 13.8

TABLE 36: Race model mean performance (WLS) over 20 models, trained with initial parameters. Influence of different decision dependent parameters.

	WLS	α	β	k	b_0	μ_{ND}	σ_{ND}	θ_{crit}
race (α, b_0, k)	1.56	Go: 1.17 Wait: 1.15	0.100	Go: 1.35 Wait: 1.28	Go: 0.425 Wait: 0.404	1.45	0.137	13.3
race (α, b_0, k, θ_{crit})	1.52	Go: 1.45 Wait: 1.05	0.098	Go: 1.12 Wait: 1.54	Go: 0.420 Wait: 0.480	1.55	0.119	Go: 13.7 Wait: 13.8

TABLE 37: Race model mean performance (WLS) over 20 models, trained with parameters of the drift-diffusion model trained for ”Go” and ”Wait” decisions. Influence of different decision dependent parameters.

zero to 2.5 seconds with a time interval of 0.05 seconds we calculated the mean RMSE value over 5 independent models (Figure 7).

We see in the results that the performance of the models is affected by the inter-judgment time until the inter-judgment time reaches a value of approximately 1.0 seconds after which the performance remains constant (Figure 7). At the inter-judgment time of 1.0 seconds, both models perform optimal. This finding suggests that the model is over parameterised or fails to correctly describe the evidence accumulation process for a longer period of time after the decision is made. The model does for example not account for the moment in time in which the decision is made or in which the oncoming vehicle has passed.

	Bias parameter	Sensitivity parameter
Model 1: DDM, $\tau = 0s$	Go: 1.83 Wait: 1.61	Go: 2.23 Wait: 3,23
Model 2: DDM, $\tau = 1.0s$	Go: 3.92 Wait: 2.02	Go: 0.269 Wait: 0.521
Model 3: Race model, $\tau = 0s$	Go: 3.14 Wait: 3,25	Go: 2.54 Wait: 0.203
Model 4: Race model, $\tau = 1.0s$	Go: 3.76 Wait: 2.96	Go: 0.257 Wait: 0.151

TABLE 38: The optimised confidence model parameters. These parameter values are used for the different presented confidence models.

Confidence model based on a general race model
Besides the four models described in the paper we made two additional confidence models, two confidence models based on the decision race model in which all model parameters are decision dependent (total of 14 + 4 free parameters). The found bias parameters and sensitivity parameters were respectively [”go”: 3.46; ”wait”: 2.87] and [”go”: 2.32; ”wait”: 0.989] for the model not allowing for post-decision evidence accumulation and respectively [”go”: 4.13; ”wait”: 2.00] and [”go”: 0.286; ”wait”: 0.614] for the model allowing for post-decision evidence accumulation for 1.0 seconds. We found that the use of this race model instead of the race model with decision dependent drift rate parameters and critical values of the generalise gap did not improve the performance, Table 39.

	RMSE
Model 1: DDM, $\tau = 0s$	0.403
Model 2: DDM, $\tau = 1.0s$	0.107
Model 3: Race model, $\tau = 0s$	0.258
Model 4: Race model, $\tau = 1.0s$	0.274
General race model, $\tau = 0s$	0.255
General race model, $\tau = 1.0s$	0.132

TABLE 39: Performance (RMSE) of the discussed confidence models.

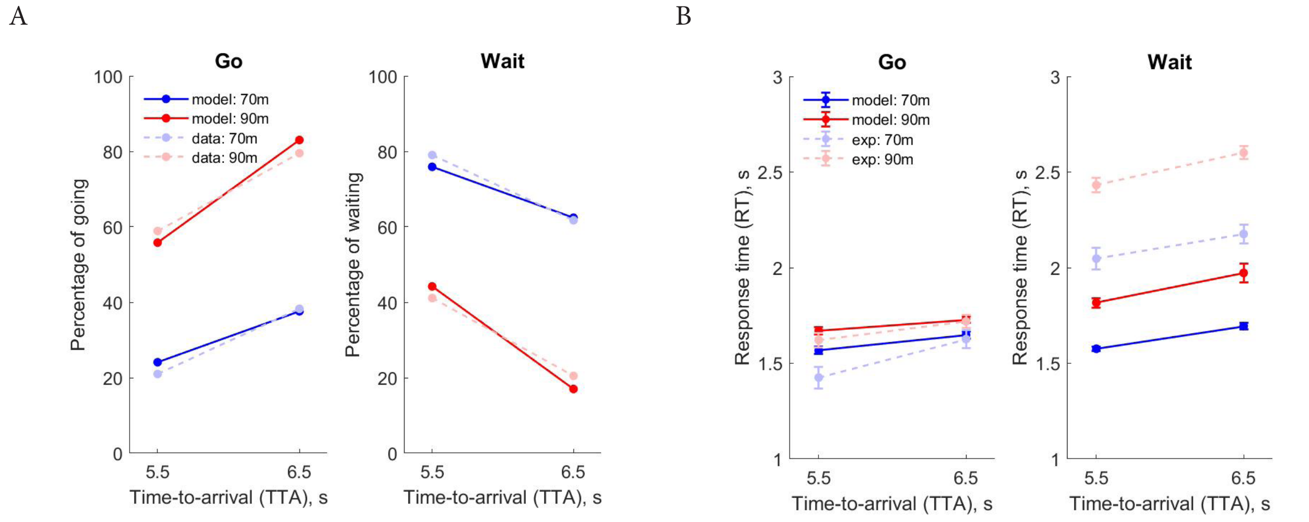

Fig. 6: Performance of drift-diffusion decision model: A) decision outcome predictions; and B) response time predictions. This model was trained with initial parameters trained for ”go” decisions.

Appendix G - Excluded Left-turns

The data analysis we present in this study is restricted to the left turn trials in which the indicated decision was conducted. We excluded all changes of mind, situations in which the participant carried out a different decision than indicated, as well as the trials in which the participants did not indicate their decision (no button press). In 3.4 % of all the decisions, changes of mind were present, of which in 83.9% of the cases the participant indicated a “go” decision and performed a “wait” decision. In 2.2% of all decisions, no button press was present. In 83.33% of these cases participants performed a “go” decision.

References

[1] Arkady Zgonnikov, David Abbink, and Gustav Markkula. Should I Stay or Should I Go? Cognitive Modeling of Left-Turn Gap Acceptance Decisions in Human Drivers. *Human Factors: The Journal of the Human Factors and Ergonomics Society*, page 001872082211445, December 2022.

[2] Roger Ratcliff and Francis Tuerlinckx. Estimating parameters of the diffusion model:approaches to dealing with contaminant reaction times and parameter variability. *Psychonomic Bulletin & Review*, 9(3):438 – 481, 2002.

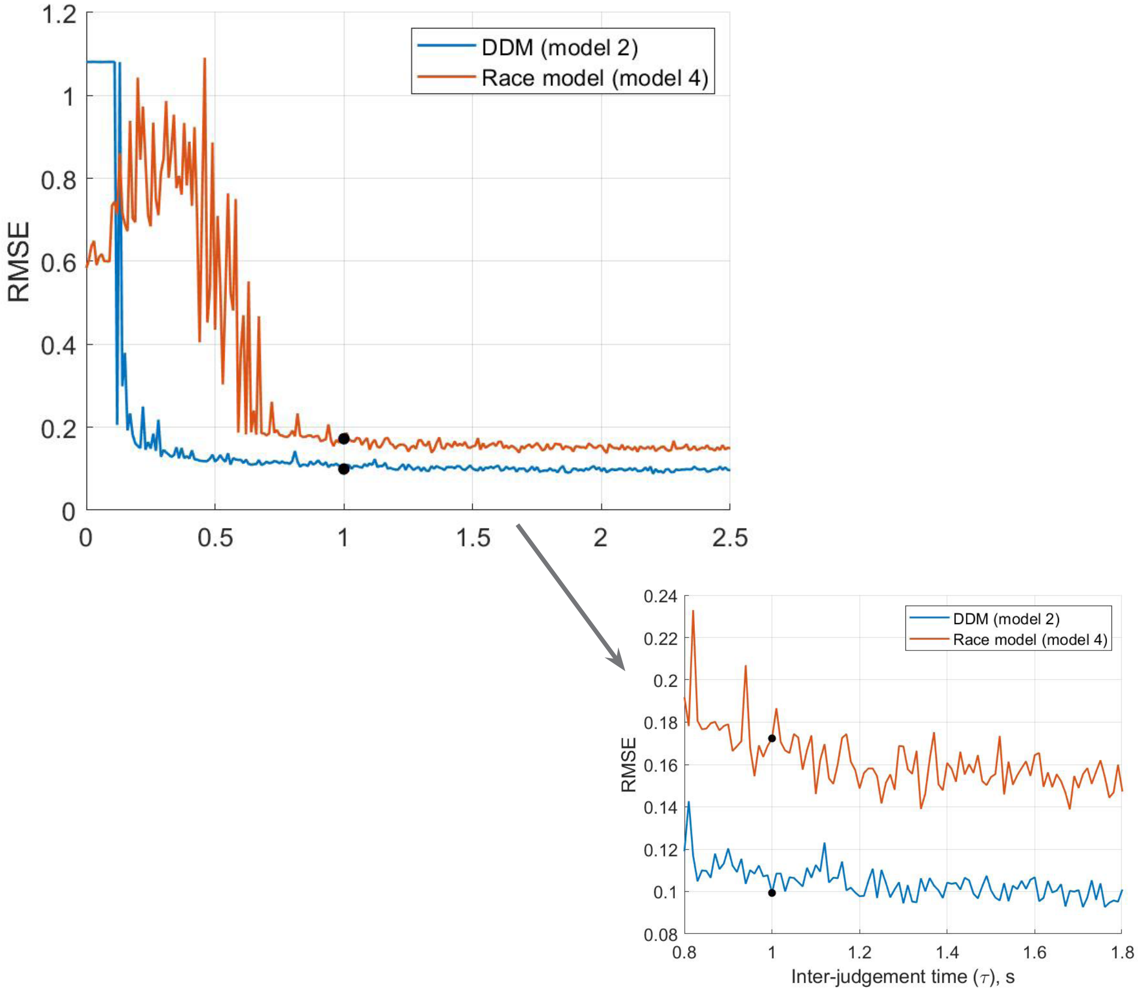

Fig. 7: Effect of different inter-judgment times on the performance (RMSE) of the confidence model based on the drift-diffusion decision model (model 2) and the confidence model based on the race model (model 4). The black point indicates the inter-judgment time used for the final confidence models (1.0 seconds)
